# Empowering Healthy Lifestyle Behavior Through Personalized Intervention Portfolios Using a Healthy Lifestyle Recommender System to Prevent and Control Obesity in Young Adults: Pilot Study Protocol from the HealthyW8 Project

**DOI:** 10.3390/jpm15120625

**Published:** 2025-12-13

**Authors:** Silvia García, Marina Ródenas-Munar, Torsten Bohn, Astrid Kemperman, Daniela Rodrigues, Suzan Evers, Elsa Lamy, María Pérez-Jiménez, Sarah Forberger, Maria Giovanna Onorati, Andrea Devecchi, Tiziana De Magistris, Jihan Halimi, Yoanna Ivanova, Boyko Doychinov, Cristina Bouzas, Josep A. Tur

**Affiliations:** 1 Foundation of the Healthy Research Institute of the Balearic Islands (IDISBA), 07120 Palma de Mallorca, Spainm.rodenas@uib.es (M.R.-M.); cristina.bouzas@uib.es (C.B.); 2 Research Group on Community Nutrition & Oxidative Stress, University of the Balearic Islands-IUNICS, 07122 Palma de Mallorca, Spain; 3 CIBEROBN (CIBER of Physiopathology of Obesity and Nutrition), Institute of Health Carlos III, 28029 Madrid, Spain; 4 Nutrition and Health Research Group, Department of Precision Health, Luxembourg Institute of Health, L-1445 Strassen, Luxembourg; torsten.bohn@lih.lu; 5 Research Group on Urban Planning and Transportation, Eindhoven University of Technology, 5612 AE Eindhoven, The Netherlands; a.d.a.m.kemperman@tue.nl (A.K.); s.evers@tue.nl (S.E.); 6 Research Centre for Anthropology and Health, Department of Life Sciences, University of Coimbra, 3000-456 Coimbra, Portugal; drdc@uc.pt; 7 Mediterranean Institute for Agriculture Environment and Development, University of Evora, 7002-554 Evora, Portugal; ecsl@uevora.pt (E.L.); maria.jimenez@uevora.pt (M.P.-J.); 8 Leibniz Institute for Prevention Research and Epidemiology-BIPS, 28359 Bremen, Germany; forberger@leibniz-bips.de; 9 University of Gastronomic Sciences, 12042 Pollenzo, Italy; m.onorati@unisg.it (M.G.O.);; 10 Instituto Agroalimentario de Aragón, IA2 (CITA-Universidad de Zaragoza), 50059 Zaragoza, Spain; tmagistris@cita-aragon.es (T.D.M.); jhalimi@cita-aragon.es (J.H.); 11 Regional Cluster North-East, 9002 Varna, Bulgariarc_ne@mail.bg (B.D.)

**Keywords:** mHealth, gamification, digital health, young adults, obesity prevention, sedentary behavior

## Abstract

**Background:** Rising obesity rates among young adults increase long-term health risks, especially cardiometabolic conditions such as type 2 diabetes mellitus. Digital interventions can offer scalable solutions to promote and support healthy behaviors by integrating personalized diet, physical activity promotion, and behavioral support. **Objective:** To assess the feasibility, user friendliness, adherence, and satisfaction of the Healthy Lifestyle Recommender System (HLRS). Secondary outcomes will include measures of metabolic health and obesity. **Methods:** A 3-month, single-arm pilot study conducted across European countries, including Bulgaria, Germany, Italy, Netherlands, Portugal, and Spain, enrolling 351 young adults (18–25 years old, BMI 18.5–29.9 kg/m^2^). The intervention includes a mobile app for meal planning (Nutrida v.1), gamified physical activity encouragement (GameBus), and real-time monitoring via a wearable smartwatch device. Primary outcomes are adherence and engagement, measured through app usage and participant feedback; secondary outcomes include anthropometry, physical activity, dietary patterns, psychological well-being, and selected biomarkers of metabolic health. **Expected Outcomes:** Improved engagement is expected to enhance lifestyle behaviors, supporting weight management and overall well-being. Findings will guide future large-scale interventions. **Conclusions:** This study will contribute to minimizing the impact of obesity in Europe.

## 1. Introduction

Young adulthood, often defined as ages 18–24 [1], represents a crucial life stage marked by increased independence, lifestyle transitions, and the establishment of long-term health behaviors. Many young adults leave their family homes to pursue education or start professional careers, which brings environmental changes and makes this age critical for weight control [2]. However, this period is also associated with a higher risk of adopting unhealthy dietary habits, reduced physical activity, and increased sedentary behaviors, contributing to weight gain and metabolic health risks [3,4,5,6,7]. In 2022, one in eight people worldwide were living with obesity; adult obesity has more than doubled since 1990. Of 2.5 billion adults aged 18 or older (43% of adults), 890 million were living with obesity (16% of the population). Given the rising obesity rates in this population [8], early and tailored interventions are essential to prevent long-term health consequences [9].

Traditional weight management strategies, such as prescribed weight-loss diets or physical activity [10,11] often fail to engage young adults due to their structured and rigid nature, as well as common barriers such as time constraints, financial limitations, and a preference for convenience over nutritional quality [12,13]. Digital health technologies, including mobile applications and wearable devices, offer a promising alternative by providing flexible, interactive, and personalized approaches to health promotion [14,15,16].

The widespread integration of technology into daily life has positioned digital health tools as a promising approach for promoting healthy behaviors and preventing chronic diseases. This is particularly relevant for young adults, who have grown up in a digital environment and are highly accustomed to using mobile applications and wearable devices. Studies have shown that adherence to digital health interventions is generally higher in young adults compared to older populations, likely due to greater technological literacy and engagement [17,18].

The HealthyW8 project, a pan-European initiative funded by the EU Horizon Calls, addresses these challenges by implementing a Healthy Lifestyle Recommender Solution (HLRS) by means of a digital platform using mobile applications. The novelty of the HealthyW8 project is its approach to weight gain through personalized and holistic recommendations, integrating key lifestyle factors such as dietary patterns, physical activity, sleep, psychological and behavioral aspects, and emotional well-being, and using behavioral nudging, gamification, and real-time feedback to enhance user engagement and adherence. Additionally, the system will use a Human Digital Twin (HDT) model, which collects data from wearable devices, self-reported inputs, and app interactions to anticipate barriers and tailor recommendations dynamically (https://www.healthyw8.eu/ accessed 21 October 2025).

Building on this evidence, the current study will evaluate the feasibility, adherence, and user satisfaction of the digital HLRS among young adults in the participating consortium countries, recognizing this life stage as pivotal for establishing lasting health behaviors. The findings will provide valuable insights to optimize digital interventions for obesity prevention in this demographic, ensuring their relevance and long-term effectiveness.

## 2. Methods

### 2.1. Study Design

This study is a prospective, multi-center, single-arm, non-controlled 3-month pilot trial conducted across several European locations including Bulgaria, Germany, Italy, Netherlands, Portugal, and Spain. It is designed as a preliminary three-month intervention (pre-post design) to test the feasibility and effectiveness of a mobile health application, with the goal of laying the foundation for a future 12-month longitudinal study with an intervention group (app) and a control group (standard recommendations). The current study design will be consistent across countries, with minor adaptations to account for local contexts. NCT07011368 is the registration number for this study on ClinicalTrials.gov (https://clinicaltrials.gov/study/NCT07011368 (accessed on 8 July 2025)).

### 2.2. Population: Inclusion and Exclusion Criteria

The pilot trial usually involves 30 young adults of both sexes per country; however, the final number of participants depends on each country, and there are a total of approximately 351 participants (Table 1). Inclusion criteria are age of 18–25 years, residence in the regions where the trials are conducted, BMI between 18.5 and 29.9 kg/m^2^ (normal weight to overweight), willingness to participate, ownership of a smartphone, and basic mobile application skills.

Exclusion criteria include individuals with obesity (BMI > 30.0 kg/m^2^), chronic conditions such as cancer, Parkinson’s disease, or other illnesses requiring specific treatments or significantly affecting normal physiology (e.g., metabolic disorders), cognitive impairments (e.g., Alzheimer’s disease), inability to live independently, adherence to specific diets, or inability to provide voluntary informed consent. However, individuals being treated for conditions such as type 2 diabetes, hypertension, or elevated blood lipids (e.g., triglycerides, cholesterol) will be included to ensure the recruitment reflects the typical demographic. If the drop-out or discontinuation rate exceeds 10%, additional participants will be recruited to maintain a minimum sample size of 20 participants per study site.

### 2.3. Participants’ Involvement

Participants who meet the inclusion criteria will be involved in setting the research question and in the design and implementation of the intervention and outcome measures. Participants will also play a central role in disseminating baseline information, which helps to motivate community involvement during and beyond the study.

### 2.4. Recruitment

Recruitment is scheduled to begin in the last semester of 2025. Strategies and specific details for each country are outlined in Table 1. Potential participants can register by phone, email, or postal mail, as well as through universities, social media campaigns, and healthcare facilities. Recruitment strategies will be adapted per country to ensure cultural and contextual relevance. Interested individuals will complete an online screening questionnaire to determine eligibility. Study personnel will organize an enrollment session to provide a brief overview of the study, distribute the participant information sheet, and obtain informed consent from each participant.

### 2.5. Intervention

The intervention was developed collaboratively by the HealthyW8 consortium and global healthcare professionals to meet the unique needs of young adults. It offers personalized features that enable participants to tailor their dietary and physical activity choices based on preferences, budget, allergies, and physical capabilities. Central to the intervention is the HLRS, which included features shown in Table 2.

Optional access to the Open Stakeholders Platform (OSP) for supplementary information and knowledge exchange among stakeholders in obesity prevention will be found at https://www.stakeholders.healthyw8.eu/.

Table 3 provides an overview of the specific tools and equipment used in each participating country for the pilot study. All tools for data and sample collection and analysis, as well as for shipping these samples to the laboratories that will analyze them (LIH and IDISBA), were previously defined and harmonized, ensuring methodological consistency while allowing for country-specific adaptations.

### 2.6. Follow-Up and Data Collection

**Figure 1:**
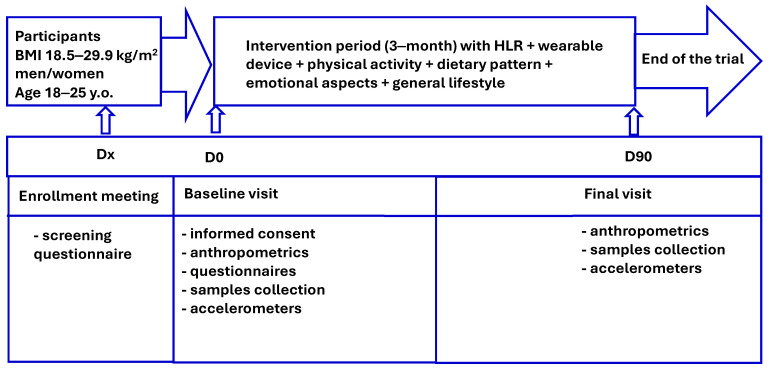
Flow-chart of the trial design and data and sample collection.

The study spans 90 days (3 months) and is structured into five phases: enrollment (Dx), baseline visit (Day 0), and final visit (Day 90) in most of the participating partners. Data collection occurs at baseline and final visits, with regular check-ins via phone calls, or in-person meetings to maintain engagement. Figure 1 summarizes the timeline and interventions at each stage. Each study phase (outlined in Table 4) is described as follows:

- Enrollment visit (Dx): Informed consent (after receiving written and verbal information about the study’s objectives, risks, and benefits), eligibility assessment (assessed based on anthropometric measurements, and screening questionnaires on general health and lifestyle), and meeting inclusion criteria.
- Baseline visit (Day 0): Anthropometric data, baseline questionnaires, biological samples (blood, urine, and saliva, where applicable), and instructions on using the HLRS platform. Recommendations will be adapted to the previously diagnosed health status, A1c levels or blood pressure of the participant. Participants will receive instructions on using the HLRS platform.
- Final visit (Day 90): Repeat assessments and questionnaires to evaluate changes over the study.

### 2.7. Outcome Measures and Data Collection

Primary outcomes focus on adherence and user experience, measured by app usage frequency/duration and wearable device data. User experience is evaluated using validated tools such as the User Experience Questionnaire [20,21].

Secondary outcomes include anthropometric measures, biological markers, physical activity, and dietary patterns, assessed through wearables and validated questionnaires, as described below and summarized in Table 4.

Anthropometric measurements: Body weight, height, waist circumference, hip and thigh circumferences will be taken, and body composition will be conducted following standardized procedures, with specific equipment varying by country. Body weight will be recorded with participants wearing light clothing and no shoes. A predetermined weight will be subtracted to account for clothing. Height will be measured to the nearest millimeter with participants standing upright and maintaining the Frankfurt Horizontal Plane. Body mass index (BMI) will be calculated as weight (kg) divided by the square of height (m^2^). Waist circumference (WC) and hip circumference (HC) will be measured in duplicate using an anthropometric tape, ensuring proper positioning and participant posture. WC will be assessed at the midpoint between the lowest rib and the iliac crest, while HC will be measured at the widest part of the hips, with the tape placed horizontally around the body. Participants will stand upright with feet together, and the average of the two measurements will be recorded for analysis [22].

Biological samples: The participants may provide saliva, urine, and blood samples. Urine samples will be collected the day after completing a 24 h Recall (24hR) to accurately assess dietary biomarkers. Additional assessments, including markers of oxidative stress and inflammation (Table 4), will be performed using commercial ELISA kits according to the manufacturer’s instructions [23,24,25].

Questionnaires: Validated questionnaires will be via REDCap [26], and include the following:

- 24hR (24-Hour Recall): Completed three times to assess dietary intake [27].
- BFI-10 (Big Five Inventory—10 Items): A personality assessment tool [28].
- IPAQ-S (International Physical Activity Questionnaire—Short Form): Measures physical activity levels [29].
- PHQ-9 (Patient Health Questionnaire—9): Evaluates symptoms of depression [30].
- PSQI (Pittsburgh Sleep Quality Index): Assesses sleep quality and disturbances [31].
- UEQ (User Experience Questionnaire): Measures user experience with digital platforms or interventions [32,33].
- WHOQOL (World Health Organization Quality of Life): Assesses quality of life across different domains [34].
- FFQ (Food Frequency Questionnaire) of 143 items: Evaluates dietary intake over a specified period [35,36,37,38].

All questionnaire data within REDCap will be securely stored on the Luxembourg Institute of Health (LIH) Cloud, with access controlled by LIH’s IT Department. Wearable device data (from Samsung or Garmin smartwatches) and GameBus information will be stored on the GameBus server, managed by the University of Eindhoven, Netherlands. Nutrida data will be stored on a secure EU-based server provided by NIUM (Amazon). Data from the HDT and calendar application will be housed on a server managed by the Luxembourg Institute of Science and Technology (LIST). Garmin smartwatches will store data directly on the device and smartphone, while Samsung data will not be stored on external company servers outside Europe or on University of Eindhoven servers. Calendar event data is securely stored on servers at LIST, Luxembourg.

## 3. Statistics

Sample Size Calculation: The study hypothesizes that the use of HLRS (≥45 min per week) will not decrease by more than 20% over the 3-month intervention period. Assuming a 30% standard deviation in usage, a 0.05 significance level, and 80% power, 20 participants would be required for a paired design. To compensate for an expected 33% attrition, 30 participants will be recruited, ensuring sufficient power. An equal distribution of males and females will be targeted to enhance the generalizability of the findings.

Handling of Missing Data and Dropout: No imputation will be performed for missing data. Participants who withdraw will be excluded from further analysis beyond their last available data. In order to maintain sample size and statistical power, replacements will be made as necessary, ensuring at least 20 participants complete the study. This method safeguards the integrity of the data and minimizes potential bias.

Data Analysis: Normality of the data will be assessed through Q-Q plots and Kolmogorov–Smirnov tests. Non-normally distributed data will be analyzed using non-parametric methods such as the Friedman test, followed by pairwise comparisons, and log transformations if appropriate. A significant level of *p* < 0.05 will be considered. Data will be analyzed using SPSS (version 25.0) and R (version 4.4.1). Since outcomes will be mainly descriptive, simple and descriptive statistics will summarize baseline participant characteristics, and changes over time will be assessed using paired t-tests, to evaluate the impact of the intervention on health outcomes.

## 4. Ethics

Compliance and Ethical Oversight: The research will be conducted in full compliance with the study protocol, good clinical practices, internal quality control systems, and the ethical guidelines set forth in the Declaration of Helsinki. The study will adhere to local regulations and legal requirements, with appropriate ethical approval obtained from relevant ethics committees in each participating country: the Balearic Islands Ethics Committee (CEIm-IB; reference: IB5752/25PI; 9 July 2025), by the Ethics Committee of University of Gastronomic Sciences, the Ethical Review Board of Eindhoven University of Technology (ERB-TU/e; ref. ERB2024BE40; 13 June 2024), the Ethics Committee of the University Bremen (ref. 2024-22; 10 September 2024), Ethics Committee of the Institute for Interdisciplinary Research of the University of Coimbra (CEIIIUC; ref. 35_ID1347; 9 September 2024), the Ethics Committee of the University of Evora (ref. 24099; 18 February 2025), the Ethics Committee of the University of Sofia, and the CITA’s Ethics Committee (CEISH; 27 November 2024).

Participant Benefits and Risks: Participants in the study will receive feedback on their health status, based on measurements previously explained. At the conclusion of the study, participants will have continued access to the HLRS mobile app features, at their own discretion and risk. The potential risks associated with this study are minimal. Aside from minor discomfort from peripheral blood draws, no invasive procedures will be carried out. Participants who experience significant medical issues, such as severe allergic reactions or high fever, will be withdrawn from the study. Likewise, individuals experiencing emotional or psychological distress will be advised to seek professional help and may be withdrawn if necessary. Given that the study includes a mobile application, there are inherent risks such as potential breaches of personal or health data security. However, these risks are considered minimal, and measures will be implemented to ensure the privacy and protection of participant information.

The safety reporting period for this study will extend from the initiation of the study until its conclusion. Any adverse events (AEs) related to the study will be monitored until they are resolved or until the study concludes, whichever occurs first. The definitions and management of adverse events are as follows:

1. Adverse Event (AE): Any untoward medical occurrence in a participant related to study procedures (e.g., blood sampling). AEs may include any unfavorable or unintended symptoms or diseases, regardless of whether they are directly caused by the study procedures.
2. Serious Adverse Event (SAE): A severe adverse event includes events that result in the following:
  - Death;
  - Life-threatening conditions;
  - Requirement for inpatient hospitalization or prolonged hospitalization;
  - Persistent or significant disability/incapacity;
  - Congenital anomalies or birth defects. Serious events may also include medical occurrences that, based on medical judgment, could jeopardize the participant’s health and require intervention to prevent one of the aforementioned outcomes.

The intensity of AEs will be graded on a scale from mild to life-threatening, as follows:

- Grade 1 (Mild): Asymptomatic or mild symptoms, no intervention needed.
- Grade 2 (Moderate): Minimal intervention required, may limit daily activities.
- Grade 3 (Severe): Requires significant intervention, may limit self-care.
- Grade 4 (Life-threatening): Urgent intervention needed.
- Grade 5 (Death): Death related to the AE.

All serious adverse events must be reported immediately to the medical principal investigator within 24 h of occurrence, who will include these events in the annual report submitted to the local ethics committees. All project partners will secure an insurance policy in accordance with local regulations to provide coverage for any injuries or damage that may occur during the clinical study.

## 5. Data Handling and Storage

Data will be handled in compliance with the GDPR. All participant data will be pseudonymized, ensuring that identifying information is securely stored separately from other data. The mapping between participant names and reference codes will be kept in a separate, secure location to maintain confidentiality. Data will be stored according to local regulations, with appropriate measures to protect it from unauthorized access, loss, or misuse, and will then be kept for an additional five years for future research purposes, in accordance with applicable legal and ethical guidelines. Participants will have the right to access, correct, or restrict the processing of their data, as well as the right to request the deletion of their data or samples, within 10 years after the study concludes. Cross-border data transfer agreements have been signed among partners to build a common database.

## 6. Dissemination Plan

The study will follow open science principles to ensure transparency, inclusivity, and accessibility. It will involve a variety of stakeholders and use participatory methods to minimize bias and enhance the relevance of the findings. Open science practices will be applied at every stage, including study design, data collection, and analysis. All publications and data will be made available through Gold Open Access, with anonymization when necessary. Results will be stored in trusted repositories like OpenAIRE and Zenodo to ensure reproducibility. The study will also include open peer review, preprints, regular ORCID updates, and share resources under Creative Commons licenses. Findings will be disseminated through publications, conference presentations, and reports to inform future health interventions and strategies.

## 7. Conclusions

It is estimated that with 200,000 HealthyW8 users, we will prevent 10,000 obesity cases/y. In the long run, the impact will be maximized through the adoption of the project’s proposed methodology, platform, and tools by as many EU institutions and entities as possible. This study will contribute to minimizing the impact of obesity in Europe.

## Figures and Tables

**Table 1:** Recruitment strategies in countries participating in the HealthyW8 young adults’ trials.

Country, Partner	Number of Participants Targeted for Enrollment	Number of Participants Required for Study Conclusion	Recruitment Strategies	Coordinating Entity	Medical Principal Investigator
Bulgaria, RCNE	30	20	Using the network of the Regional Cluster North-East, word-of-mouth	RCNE	No
Germany, BIPS/DFKI	30	15	Word-of-mouth, flyer, press release, Instagram	BIPS	No
Italy	101	52	Flyers, word-of-mouth, conferences, social media, institutional website, local press	USG	A medical nutritionist involved in the screening
Netherlands	40	20	Flyers and posters, institutional website and newsletter, social media, word-of-mouth	TU/e	No
Portugal	30	15	Flyers, word-of-mouth, conferences, social media, institutional website, local press	CIAS, UC	No
Portugal	30	15	Flyers, word-of-mouth, conferences, social media, institutional website, local press	UEV	No
Spain	60	25	Flyers, word-of-mouth, conferences, social media, institutional website, local press	CITA	No
Spain	30	20	Word-of-mouth, newspaper advertisement, flyers, physicians, and other healthcare professionals.	IDISBA	Yes

Abbreviations: RCNE: Regional Cluster North-East; BIPS GmbH/Deutsches Forschungzentrum fur Kunstliche Intelligenz GmbH; CIAS, UC: Research Center for Anthropology and Health of the Universidade de Coimbra; CITA: Agro-food Technological Center of Aragon; USG: Universitá Degli Studi di Scienze Gastronomiche; TU/e: Eindhoven University of Technology; UEV: Universidade de Évora; CITA: Centro de Investigación y Tecnología Agroalimentaria de Aragón; IDISBA: Foundation of Institute of Health Research of the Balearic Islands.

**Table 2:** Features included in the intervention through the HLRS.

The Gamebus Application	Promotes healthy lifestyle behavior, such as physical activity (e.g., walking and step counting), diet (e.g., preparing healthy meals, increasing the consumption of fruits and vegetables), or social participation (e.g., participating in cultural activities). The app uses gamification techniques (e.g., earning points, unlocking levels, competing on leaderboards) to motivate engagement. Developed by the Technical University of Eindhoven, GameBus serves as a prototype health data management platform, enhancing user engagement through mobile health (mHealth) platforms [19]. It includes an REST API based on Java Spring, an open-source web app frontend (using Ionic), and a restricted interface for smartwatches. Additional information can be found at the following website: https://blog.gamebus.eu/.
The Nutrida Application and Recipes	Provides personalized weekly meal plans, tailored to preferences, budget, allergies, and caloric requirements. The meal recommendation system is based on the Active Assisted Living (AAL) project, LIFANA, and has been further developed by the Nutrida app, created by NIUM, a University of Luxembourg spin-off (Esch-sur Alzette, Luxembourg), in collaboration with the Luxembourg Institute of Science and Technology (LIST) and the European Federation of the Association of Dieticians (EFAD), which integrates regional recipes into the app. Obtained data will be stored in secure Amazon servers in Europe, subcontracted by NIUM.
Wearable Device	Participants use either the Samsung Galaxy Active 2 smartwatch or the Garmin Vivosmart 5 fitness tracker to monitor 24 h movement, step count, sleep, pulse, and heart rate. The Samsung Galaxy Activity 2 smartwatches do not collect sleep data; also, heart rate and pulse are not measured continuously, and it can collect emotional states via the experience sampling method (as well as GPS tracking). Both devices comply with General Data Protection Regulation (GDPR), ensuring data privacy. The final choice of device will depend on the study’s specific requirements, as both collect similar data.
Samsung Galaxy Watch Active 2 Experiencer or Garmin Vivosmart 5 App	Data collected through Samsung/Garmin App will be stored, ensuring compliance with GDPR and utilize FHIR (Fast Healthcare Interoperability Resources) for FAIR (Findable, Accessible, Interoperable, and Reusable) data management.
Actigraph Accelerometers	Participants will be required to wear an ActiGraph accelerometer (ActiGraph wGT3X-B; ActiGraph LLC, Pensacola, FL, USA) continuously for nine consecutive days to objectively assess their physical activity levels and sedentary behavior. This device will provide detailed data on movement patterns, energy expenditure, and sleep–wake cycles, allowing for a comprehensive analysis of participants’ activity and rest periods. To ensure data accuracy, participants will be instructed on proper device placement and usage.
Calendar Web Application	Developed by EU HealthyW8 partner VirTech (Bulgaria), this application enables the study leader to schedule local events and inform participants. The event data is securely stored on servers at LIST, Luxembourg.
REDCap (Research Electronic Data Capture), VanDerBilt University (USA)	Used for completing intervention questionnaires. Additional information can be found at the following website: https://project-redcap.org/. The event data is securely stored on servers at LIST, Luxembourg.

**Table 3:** Tools used by each participating country in the young adults’ pilot study.

Country, Partner	Bulgaria, RCNE	Germany, BIPS/DFKI	Italy, USG	Netherlands, TU/e	Portugal, UC	Portugal, UEV	Spain, CITA	Spain, IDISBA
Length of intervention (months)	3	3	1.5	1–2	3	3	3	3
Length of run-in time	6	6	6	1	2	2	2	2
Gamebus	Yes	Yes	Yes	Yes	Yes	Yes	Yes	Yes
Nutrida	Yes	Yes	Yes	Yes	Yes	Yes	Yes	Yes
Wearable	Garmin	Samsung	Garmin	Samsung	Samsung	Garmin	Garmin	Garmin
Experiencer	No	No	No	Yes	Yes	No	No	No
Accelerometer	Yes	Yes	No	No	No	Yes	No	Yes
Calendar	Yes	Yes	Yes	No	No	Yes	Yes	Yes
Questionnaires	Yes	Yes	Yes	Yes	Yes	Yes	Yes	Yes
Biological samples and measures	Yes	No	No	No	No	Yes	No	Yes

Abbreviations: RCNE: Regional Cluster North-East; BIPS/DFKI: Leibniz-Institut fur Praventionsforschung und Epidemiologia-BIPS GmbH/Deutsches Forschungzentrum fur Kunstliche Intelligenz GmbH; USG: Universitá Degli Studi di Scienze Gastronomiche; TU/e: Eindhoven University of Technology; UC: Universidade de Coimbra; UEV: Universidade de Évora; CITA: Centro de Investigación y Tecnología Agroalimentaria de Aragón; IDISBA: Fundació Institut d’Investigació Sanitària de les Illes Balears.

**Table 4:** Measures included in pilot studies of each team.

Marker Class	Marker	Bulgaria, RCNE	Germany, BIPS/DFKI	Italy, USG	Netherlands, TU/e	Portugal, UC	Portugal, UEV	Spain, CITA	Spain, IDISBA
Anthropometrics	Height, weight, BMI	Yes	No	Yes	Yes	Yes	Yes	Yes	Yes
	Waist and hip circumference and ratio	No	No	Yes	No	Yes	Yes	No	Yes
	%body fat	No	No	Yes	No	Yes	Yes	No	Yes
	Visceral fat	No	No	No	No	Yes	Yes	No	Yes
	Thigh circumference	No	No	Yes	No	Yes	Yes	No	Yes
Clinical	Heart frequency	No	No	No	No	Yes	Yes	No	Yes
	Blood pressure (systolic and diastolic)	No	No	No	No	Yes	Yes	No	Yes
PA	Accelerometer	Yes	No	No	No	Yes	Yes	No	Yes
	IPAQ	Yes	Yes	Yes	Yes	Yes	Yes	Yes	Yes
Fitness	Hand grip strength, step-up test	No	No	No	No	No	No	No	No
Dietary patterns	FFQ	Yes	Yes	Yes	No	Yes	Yes	Yes	Yes
	24 h recall	Yes	Yes	Yes	No *	Yes	Yes	Yes	Yes
	Mediterranean eating patterns (MEDAS-14) **	No	No	Yes	Yes	Yes	Yes	Yes	Yes
General questionnaires	Quality of life (WHOQOL-Bref 26 items) **	Yes	Yes	Yes	Yes	Yes	Yes	No	Yes
	UEQ+ (26 items)	Yes	Yes	No	Yes	Yes	Yes	Yes	Yes
	Depression (PHQ-9; nine items) **	Yes	Yes	Yes	No	Yes	Yes	No	Yes
	Sleeping patterns (e.g., Pittsburgh Sleep Quality Index) **	No	Yes	Yes	No	Yes	Yes	No	Yes
	Intrinsic motivation (IMI seven items), **	Yes	No	No	Yes	Yes	Yes	No	Yes
	Smoking (Fagerstrom six items) **	No	Yes	Yes	No	No	No	No	No
	Alcohol intake (Audit 10 items) **	No	Yes	Yes	No	No	No	No	No
	Personality traits (BFI-10 scale 10 items) **	Yes	No	Yes	Yes	Yes	Yes	Yes	Yes
Biomarkers, inflammation	Cytokines (adiponectin, TNF-α, IL6, IL1-β, CRP)	No	No	No	No	No	No	No	Yes
Biomarkers, oxidative stress	F2- isoprostanes, malondialdehyde, DNA/RNA breakdown products, antioxidant activity (FRAP, ABTS, MDA)	No	No	No	No	No	No	No	Yes
Biomarkers of nutrient intakes	Blood cell counts, albumin, prealbumin,	No	No	No	No	Yes	Yes	No	Yes
Glucose metabolism	HbA1c and fasting blood glucose, insulin, HOMA-IR	No	No	No	No	Yes	Yes	No	Yes
Lipid profile	Total cholesterol, HDL-C, LDL-C, triglycerides	No	No	No	No	Yes	Yes	No	Yes
Urinary markers	Uric acid, urinary creatinine, urinary sodium,	No	No	No	No	Yes	Yes	No	Yes
Saliva	Cortisol	No	No	No	No	Yes	Yes	No	Yes
HLRS	Nutrida-app: Personalized meal recommender plan (based on age, gender, PA, dietary restrictions, culinary/ cultural personal preferences and local dishes, budget…)	Yes	Yes	Yes	Yes	Yes	Yes	Yes	Yes
	GameBus: Application that promotes healthy lifestyle through games and tasks linked to Nutrida and smartwatches	Yes	Yes	Yes	Yes	Yes	Yes	Yes	Yes
	Smartwatch: To register step count, pulse, sleep quality	Yes	Yes	Yes	No	Yes	Yes	No	Yes
	Calendar function to schedule social events for the participants	Yes	Yes	Yes	No	No	No	Yes	No
	UEQ+ (26 items)	Yes	Yes	No	Yes	Yes	Yes	Yes	Yes
Open Stakeholder Platform	Health video clips, recipes, lifestyle recommendations	No	No	Yes	Yes	Yes	Yes	No	Yes

* It was performed in a separate study. ** Self-applied questionnaires.

## Data Availability

There are restrictions on the availability of data for this trial, due to the signed consent agreements around data sharing, which only allow access to external researchers for studies following the project purposes. Researchers wishing to access the trial data used in this study can make a request to pep.tur@uib.es.
